# Destabilized Passivation Layer on Magnesium-Based Intermetallics as Potential Anode Active Materials for Magnesium Ion Batteries

**DOI:** 10.3389/fchem.2019.00007

**Published:** 2019-01-23

**Authors:** Masaki Matsui, Hiroko Kuwata, Daisuke Mori, Nobuyuki Imanishi, Minoru Mizuhata

**Affiliations:** ^1^Department of Chemical Science and Engineering, Kobe University, Kobe, Japan; ^2^Department of Chemistry for Materials, Mie University, Tsu, Japan

**Keywords:** intermetallics, anode, passivation layer, hydrolysis, bond-valence sum mapping, Mg^2+^ diffusion pathway

## Abstract

Passivation of magnesium metal anode is one of the critical challenges for the development of magnesium batteries. Here we investigated the passivation process of an intermetallic anode: Mg_3_Bi_2_ synthesized by solid-state and thin film process. The Mg_3_Bi_2_ composite electrode shows excellent reversibility in magnesium bis(trifluoromethansulfonylamide) dissolved in acetonitrile, while Mg_3_Sb_2_, which has same crystal structure and similar chemical properties, is electrochemically inactive. We also fabricated the Mg_3_Bi_2_ thin film electrodes, which show reversibility with low overpotential not only in the acetonitrile solution but also glyme-based solutions. Surface layer corresponding to the decomposed TFSA anion is slightly suppressed in the case of the Mg_3_Bi_2_ thin film electrode, compared with Mg metal. Comparative study of hydrolysis process of the Mg_3_Bi_2_ and the Mg_3_Sb_2_ suggests that the both intermetallic anodes are not completely passivated. The bond valence sum mapping of the Mg_3_Bi_2_ indicates that the fast Mg^2+^ diffusion pathway between 2*d* tetrahedral sites is formed. The electrochemical properties of the Mg_3_Bi_2_ anode is mainly due to the less passivation surface with the fast Mg^2+^ diffusion pathways.

## Introduction

Beyond Li-ion batteries have been widely investigated last decade (Larcher and Tarascon, 2015). Alternative anode active material using electrochemical deposition-dissolution process of less-noble metal is one strategy for the development of high-energy battery system (Lin et al., 2017). Among various choices of the less-noble metal anodes, magnesium is one of the potential candidates as the high-energy anode active material, because the volumetric capacity: 3,800 mAh cm^−3^ is 1.9 times higher than that of lithium metal, and no dendritic growth during the deposition process (Matsui, 2011). On the other hand, the surface of the magnesium metal is easily passivated in conventional electrolyte solutions such as magnesium perchlorate dissolved in propylene carbonate (Lu et al., 1999). Therefore, the choice of the electrolyte solutions for rechargeable magnesium batteries is very limited. Typically the organohaloaluminate electrolyte solutions, which are widely studied by Aurbach et al. in early 2000s (e.g., Aurbach et al., 2000, 2003, 2007), show highly reversible deposition/dissolution of magnesium metal, however since these electrolyte solutions contain halides: Cl^−^ or Br^−^, the potential window of the organohaloaluminate electrolyte solutions is limited. In addition the corrosive properties of the halides initiate the dissolution of the cathode current collector at high cell voltage >2.5 V (Muldoon et al., 2012). Therefore, finding halide-free electrolyte solutions has been standing in the center of the electrolyte development for high-voltage magnesium batteries. Recently, Tutusaus et al. reported a new class of the magnesium salt having boron cluster anion monocarborane CB_11_${\text{H}}_{12^{-}}$ dissolved in glymes showed reversible deposition/dissolution of magnesium without corrosive properties (Tutusaus et al., 2015). A fluorinated alkokyborate-based electrolyte: magnesium hexafluoroisopropylaluminate (Mg[B(hfip)_4_]_2_) also shows excellent reversibility without the corrosive properties (Zhao-Karger et al., 2017). Even with the excellent electrochemical properties of these new class of the electrolytes, the oxidation stabilities of these solutions are still limited up to 3.5 V vs. Mg due to the ether-based solvents.

Our group has been working on intermetallic anodes such as Mg_3_Bi_2_ and Mg_2_Sn, because the intermetallic anodes are compatible with a conventional electrolyte solution: magnesium bis(trifluoromethansulfonylamide) (Mg(TFSA)_2_ ) dissolved in acetonitrile(AN) (Arthur et al., 2012; Singh et al., 2013). Despite the high equilibrium potential and the low specific capacity of the Mg_3_Bi_2_, the compatibility against wide variety of the electrolyte solutions could become an advantage for the development of a battery system. Moreover, since the bismuth is very heavy element, the volumetric capacity of the Mg_3_Bi_2_: 1906 mAh cm^−3^ is still comparable value to the theoretical capacity of lithium metal. Therefore, we think the “Mg-ion” battery using the intermetallic anode could be a more realistic direction to develop a practical battery system.

Even with the several reports concerning the Mg_3_Bi_2_ as the anode active material for Mg-ion batteries, there still remains room to understand why the Mg_3_Bi_2_ shows the compatibility with various electrolytes solutions. Also the fast reaction kinetics of the Mg_3_Bi_2_ among various Mg-based intermetallic anodes, are still under investigation. In the present study, we attempted to understand the origin of the wide compatibility and the fast kinetics of the Mg_3_Bi_2_ as the alternate anode active material for magnesium batteries. We investigated the electrochemical properties of the Mg_3_Bi_2_ composite electrodes, synthesized by a conventional solid-state process. In order to conduct comparative studies, another intermetallic anode: Mg_3_Sb_2_ was also investigated, because of its structural and chemical similarities to the Mg_3_Bi_2_. The Mg_3_Sb_2_ has same crystal structure to the Mg_3_Bi_2_, in both magnesiated and demagnesiated phases. In addition the binary phase diagram of Mg-Sb system shows that the expected electrochemical reaction is a two-phase reaction of Mg_3_Sb_2_ and Sb, which is exact same as the two phase reaction in the Mg-Bi system. We also fabricated Mg metal and Mg_3_Bi_2_ thin film electrodes using rf-sputtering coater to investigate the correlation between the surface layer and the overpotential of the magnesium metal and the Mg_3_Bi_2_. The surface layer on the magnesium metal and the Mg_3_Bi_2_ thin films were characterized using X-ray photoelectron spectroscopy (XPS). In order to evaluate the stability of the surface layer on the intermetallic anodes, the hydrolysis process of the Mg_3_Bi_2_ and the Mg_3_Sb_2_ were investigated. The Mg^2+^ diffusion pathways in the Mg_3_Bi_2_ and the Mg_3_Sb_2_ were estimated by bond-valence sum (BVS) mapping (Adams, 2006) to discuss the reaction kinetics of these two intermetallic anodes.

## Materials and Methods

### Synthesis and Characterization

The Mg_3_Bi_2_ and the Mg_3_Sb_2_ powders were synthesized by a conventional solid-state reaction from stoichiometric amount of magnesium powder (99.8%, Alfa Aesar) and bismuth powder (99.999%, Alfa Aesar) or antimony powder (99.999%, Alfa Aesar) with 10% excess magnesium. The metal powders were thoroughly mixed using an agate mortar and pestle. Subsequently the mixture of the materials were mechanically milled using a planetary ball mill (PL-7, Fritsch) with ZrO_2_ grinding bowls and balls, for 8 h at 400 rpm. The milled powders were pressed into pellets and calcined at 550°C for 5 h to obtain the single phase of the intermetallics. The calcined pellets were ground and sieved before the preparation of the electrode slurry. In order to avoid the oxidation of the materials, all the synthesis procedures were carried out under inert atmosphere.

The Mg_3_Bi_2_ and the magnesium metal thin films were also fabricated to characterize the passivation layer. The thin film fabrication was carried out using a magnetron sputtering coater (Pascal) equipped with 2″ of magnesium and bismuth target. Since the load-lock is directly connected to the argon-filled glove box, the thin films can be prepared without air exposure. We employed a copper foil as substrate of the thin film electrode. The native oxide layer of the copper substrate was removed by sputter etching at 20 W for 20 min. The Mg_3_Bi_2_ thin films were fabricated by co-sputtering process of magnesium and bismuth. The rf power was 50 and 20 W for the magnesium and the bismuth respectively. The sputtering duration was 15 min. The substrate was heated at 200°C and rotated at 10 rpm to obtain uniform coatings. Thin films of the magnesium metal were also fabricated almost same sputtering condition above. The sputtering duration is increased to 20 min without the sputtering of the bismuth. The thickness of the Mg_3_Bi_2_ thin film is ≈500 nm.

The synthesized powders and the thin films were characterized using X-ray diffractometer (D8 ADVANCE, Bruker) equipped with Cu-Kα radiation. The sample preparation was carried out in the argon-filled glove box < 1.0 ppm of moisture and oxygen, using an airtight sample holder to avoid the air exposure of the samples during the measurement. The structural refinement for the powder samples was carried out using RIERAN-FP ver. 2.84 (Izumi and Momma, 2007) and the crystal structures were drawn by VESTA 3.4.4 (Momma and Izumi, 2011). We also performed the BVS mapping to visualize the Mg^2+^ ion diffusion pathways, using PyAbstantia ver. 0.7 (Nishimura, 2017).

### Electrochemistry

The electrode slurry was prepared by mixing the 80 wt% of the Mg_3_Bi_2_ or Mg_3_Sb_2_ powder, 10 wt% of acetylene black (TIMCAL), and 10 wt% of polyimide binder (Toray) dissolved in anhydrous NMP. The mixed slurry was coated on the copper foil and dried at 80°C under vacuum and pressed. Subsequently the electrode were heated at 350°C under argon flow for the thermal imidization of the binder. The loading level of the Mg_3_Bi_2_ and Mg_3_Sb_2_ are within the range 2.8–3.3 mg cm^−2^. The Mg_3_Bi_2_ thin film electrodes were used without further treatment.

Conventional three-electrode cells were employed for the electrochemical measurement. The composite/thin film electrodes were used as the working electrodes. The counter electrode was a platinum or magnesium foil. The reference electrode had a silver wire immersed in 0.05 mol L^−1^ AgNO_3_(Wako) in acetonitrile: AN (Kishida) solution with two liquid junctions with microporous glass membranes to avoid contamination of the Ag^+^ ion into the electrolyte solution. A 1.0 mol L^−1^ magnesium bis(trifluoromethanesulfonyl)amide:Mg(TFSA)_2_ in AN solution (Kishida) was used as the electrolyte solution. We also prepared glyme-based electrolyte solutions. The Mg(TFSA)_2_ salt (Kishida) was vacuum dried at 170°C for 8 h. The glyme-based solvents monoglyme: G1 (Kishida) and butylmethyltriglyme:BuMeG3 (Wako) were dried over activated molecular sieve (5A) for 36 h until the water content reaches < 15 ppm. Subsequently the Mg(TFSA)_2_ was dissolved in the solvents to use as the electrolyte solutions. The water content of the solvents and solutions were measured by Karl Fischer titration method (Mitsubishi Chemical Analytics).

### Surface Analysis

In order to characterize the passivation layer at the surface of the Mg_3_Bi_2_ thin film electrode, we conducted X-ray photo electron spectroscopy: XPS (ESCA3400, Shimadzu) with Mg-Kα radiation. As a comparison, the XPS spectra for a magnesium metal thin film was also measured. The Mg_3_Bi_2_ and the magnesium metal thin films were immersed in the BuMeG3 solution for 24 h, right after the sputtering process. Subsequently the thin films were rinsed with monoglyme(G1) to remove the BuMeG3 solutions. The emission current and the acceleration voltatge of the X-ray gun is 20 mA and 10 kV respectively. Argon ion etching, with 20 mA of emission current and 2 kV of acceleration voltage, was carried out to take the depth profile.

## Results and Discussions

### Electrochemical Properties of the Mg_3_Bi_2_ and Mg_3_Sb_2_ Composite Electrode

**Figure 1:**
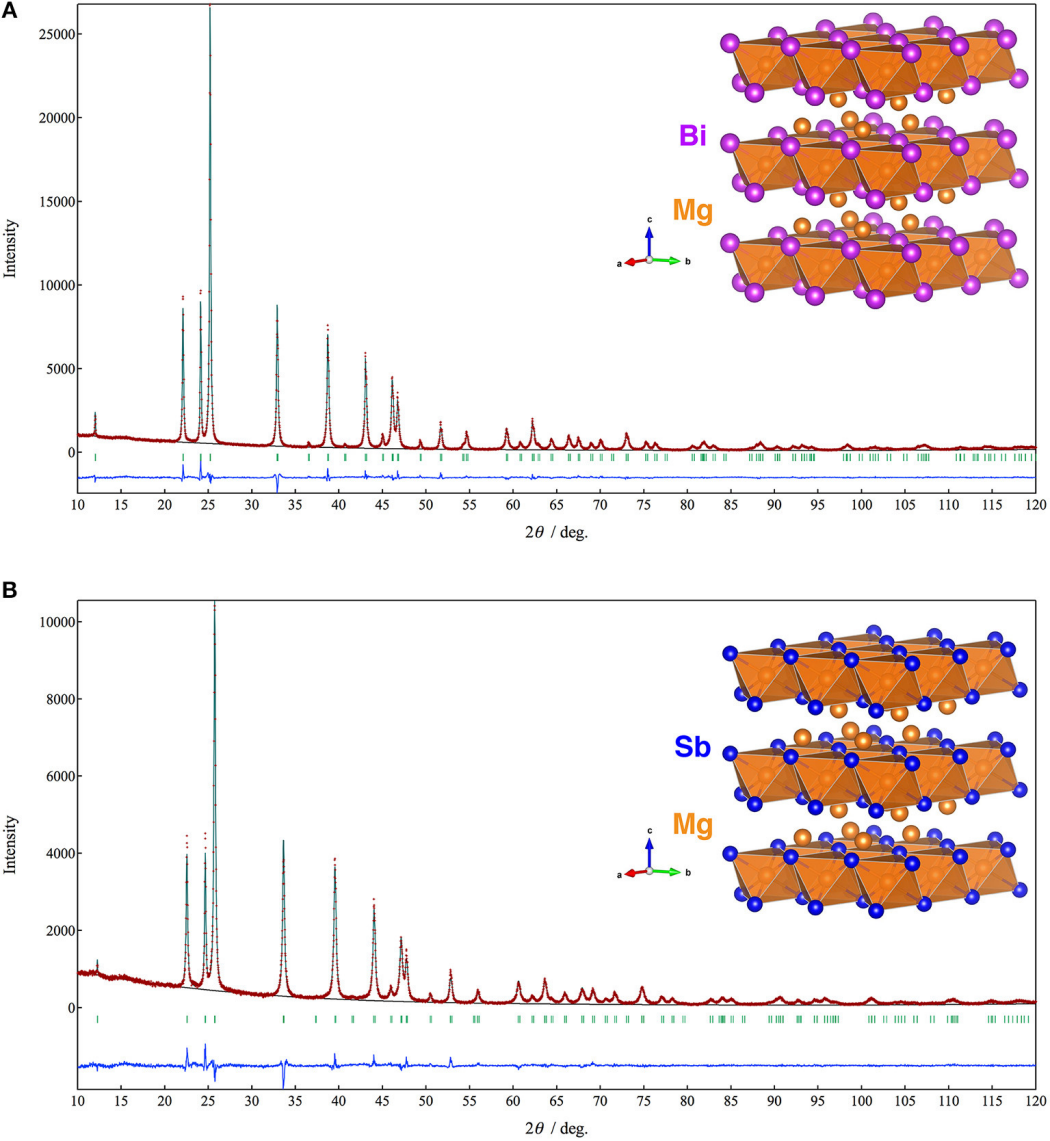
Powder XRD patterns and the crystal structures of **(A)** Mg_3_Bi_2_ and **(B)** Mg_3_Sb_2_. The brown symbols are measured value of the PXRD data, the dark green line is the simulated pattern. The light green marks represents the peak positions and the blue line is corresponding to the residue.

Figure 1 shows XRD patterns and Rietveld refinement results of the Mg_3_Bi_2_ and Mg_3_Sb_2_. Both of the diffraction patterns are assigned to A-type rare earth structure with trigonal *P*$\bar{3}$*m*1 space group. The refinement yields the trigonal lattice with *a* = 4.65349(5) Å and *c* = 7.38997(9) Å for Mg_3_Bi_2_ and *a* = 4.55963(10) Å and *c* = 7.22933 Å for Mg_3_Sb_2_ respectively. The magnesium occupies 1*a* site and half of 2*d* sites. The bismuth or antimony occupies another half of 2*d* sites. Both refinement results are in good agreement with reported data (Ahmadpour et al., 2007; Liu et al., 2017). Summary of the refinement results are shown in Tables 1, 2. The crystal structure of both the Mg_3_Bi_2_ and the Mg_3_Sb_2_ can be described as a layered structure of hexagonal close-packed (h.c.p) bismuth array with Mg atoms located at the octahedral voids in every two bismuth c.p. layers, and the tetrahedral voids in the another two bismuth c.p. layers.

**Table 1:** Rietveld refinement result of Mg_3_Bi_2_.

**(A) Crystallographic Data of Mg**_****3****_**Bi**_****2****_						
**Crystal system, Space group**					**Trigonal, *P*$\bar{3}$*m*1(No. 164)**	
*a*/Å					4.65349(5)	
*c*/Å					7.38997(9)	
Cell volume/Å^3^					138.5899(28)	
*R*_wp_					6.190	
*R*_p_					4.746	
GoF					1.4560	
*R*_b_					1.567	
**(B) Fractional Atomic Coordinates, Occupancies and**						
**Isotropic Displacement**						
**Site**	**Wyckoff**	***x***	***y***	***z***	**occ**.	***B*****(Å)**
Mg(1)	1*a*	0	0	0	1	1.5
Mg(2)	2*d*	0.33333	0.66667	0.63187 (61)	1	1.5
Bi(1)	2*d*	0.33333	0.66667	0.22473 (10)	1	0.9

**Table 2:** Rietveld refinement result of Mg_3_Sb_2_.

**(A) Crystallographic Data of Mg**_****3****_**Sb**_****2****_						
**Crystal system, Space group**					**Trigonal, *P*$\bar{3}$*m*1(No. 164)**	
*a*/Å					4.55963(10)	
*c*/Å					7.22933(16)	
Cell volume/Å^3^					130.1629(51)	
*R*_wp_					7.155	
*R*_p_					5.501	
GoF					1.8635	
*R*_b_					1.906	
**(B) Fractional Atomic Coordinates, Occupancies, and Isotropic**						
**Displacement**						
**Site**	**Wyckoff**	***x***	***y***	***z***	**occ**.	***B*****(Å)**
Mg(1)	1*a*	0	0	0	1	1.2
Mg(2)	2*d*	0.33333	0.66667	0.63501 (51)	1	1.2
Bi(1)	2*d*	0.33333	0.66667	0.22979 (15)	1	0.9

**Figure 2:**
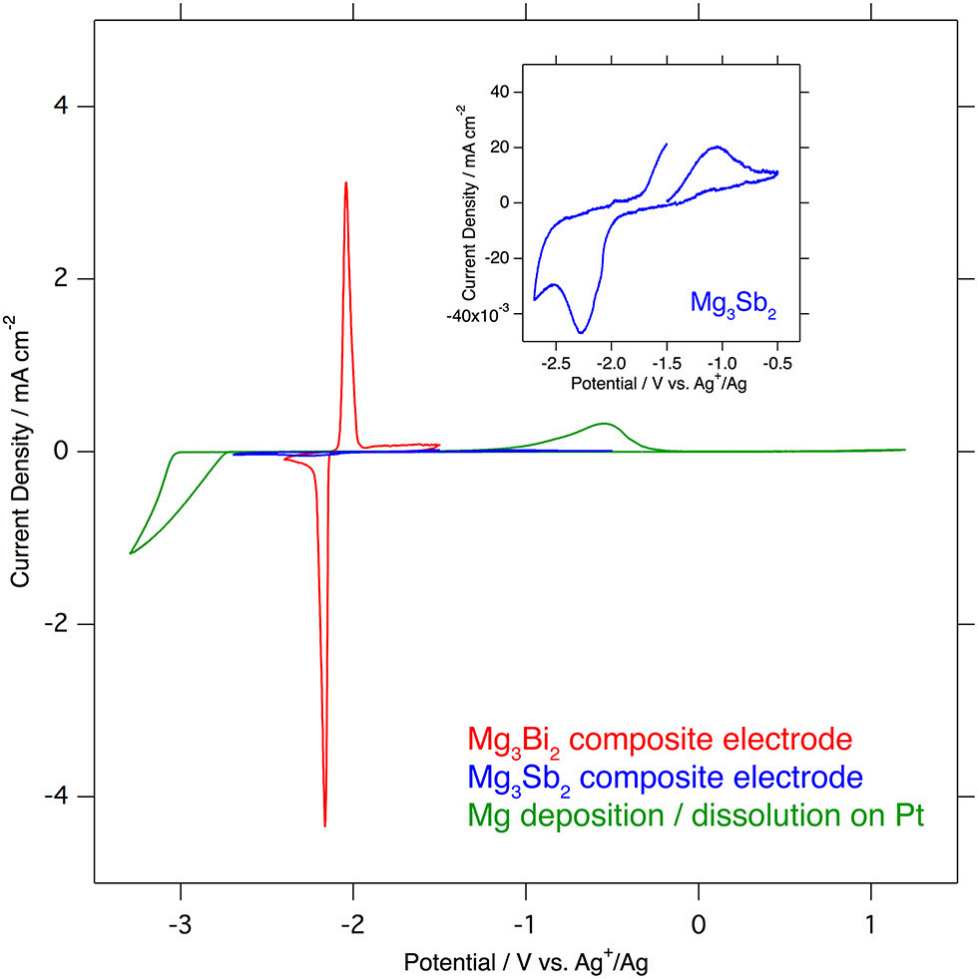
Comparison of cyclic voltammograms of Mg_3_Bi_2_ composite electrode (red), Mg_3_Sb_2_ (blue) in 1.0 mol L^−1^ Mg(TFSA)_2_ in acetonitrile and Mg deposition/dissolution in 0.5 mol L^−1^ Mg(TFSA)_2_ in BuMeG3 (green). The sweep rates of the voltammetry are 0.1 mV s^−1^ for Mg_3_Bi_2_ and Mg_3_Sb_2_, and 1.0 mV s^−1^ for Mg deposition/dissolution.

Even though the crystal structures of the Mg_3_Bi_2_ and the Mg_3_Sb_2_ are very similar except the slight difference of the lattice constants, the electrochemical properties of these two compounds are significantly different. The cyclic voltammograms of the Mg_3_Bi_2_ and the Mg_3_Sb_2_ composite electrodes in 1.0 mol L^−1^ Mg(TFSA)_2_ in AN solution, overlapped with a cyclic voltammogram of the deposition/dissolution process of magnesium metal in 0.5 mol L^−1^ Mg(TFSA)_2_ in BuMeG3 solution, are shown in Figure 2. The Mg_3_Bi_2_ shows very sharp reversible peaks corresponding to demagnetization/magnetization process at −2.1 V vs. Ag^+^/Ag. Even though the sweep rate of the cyclic voltammogram is faster than reported data, the overpotential between the anodic and cathodic reaction is almost same as previous one: ~80 mV. It suggests that the overpotential is not corresponding to the reaction kinetics, but thermodynamic properties, such as solvation/desolvation energy or phase transition of the solid phase. In addition, the overpotential of the Mg_3_Bi_2_ is much lower than that of the deposition/dissolution process of the magnesium in the BuMeG3 solution. Hence the Mg_3_Bi_2_ obviously has some advantages as a practical battery anode in the aspect of energy efficiency, besides its less capacity and higher equilibrium potential compared with magnesium metal anode. The expanded cyclic voltammogram of the Mg_3_Sb_2_ is also shown in the inset of Figure 2. Reversible current around −2.05 V vs. Ag^+^/Ag is observed, and another anodic current appeared at −1.7 V vs. Ag^+^/Ag and above. It indicates the Mg_3_Sb_2_ also has some reversibility at slightly higher equilibrium potential of Mg_3_Bi_2_, the observed current is however negligible compared with Mg_3_Bi_2_. We suspect only the surface of the Mg_3_Sb_2_ particle is electrochemically active in the composite electrode.

### Electrochemical Properties of the Mg_3_Bi_2_ Thin Film Electrodes

**Figure 3:**
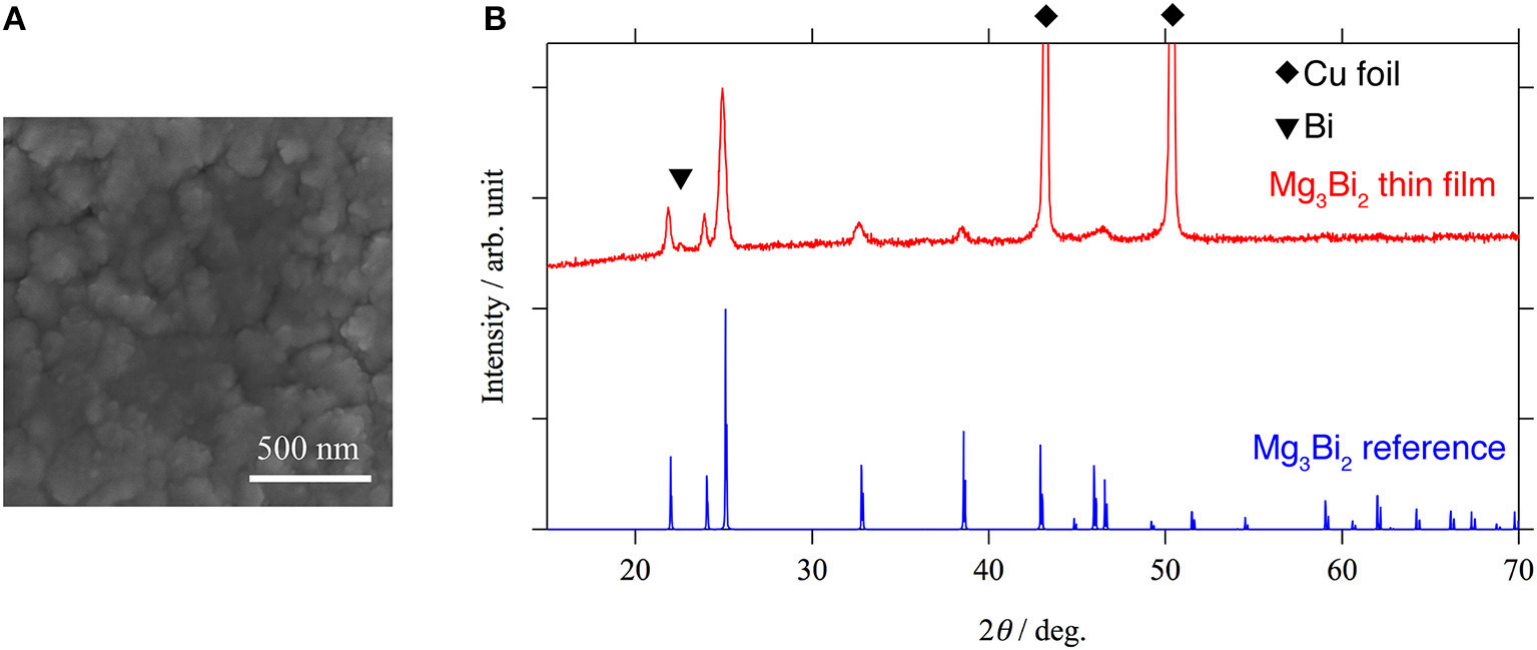
An SEM image **(A)** and an XRD pattern **(B)** of Mg_3_Bi_2_ thin film. The small peak at 22.6° of 2θ corresponds to 003 reflection of bismuth. Two strong peaks observed at 44.9 and 49.9° of 2θ are corresponding to the copper substrate. All the other peaks match with the reference pattern of Mg_3_Bi_2_ (ICDD PDF 04-003-6859).

In order to investigate the correlation between the electrochemical properties of the Mg_3_Bi_2_ and the surface layer, Mg_3_Bi_2_ thin film electrodes were fabricated by magnetron sputtering process. An SEM image and an XRD pattern of the Mg_3_Bi_2_ thin film are shown in Figure 3. The SEM image shows a polycrystalline Mg_3_Bi_2_ thin film having 200–300 nm of secondary particles. The diffraction pattern of the Mg_3_Bi_2_ thin film well matches with the powder pattern discussed in the previous section. Since the fabrication process does not have post annealing process, the Mg_3_Bi_2_ thin film has slightly poor crystallinity. The small peak observed at 22.6° of 2θ is corresponding the 003 reflection of the bismuth remained as impurity phase. The peak of 012 reflection of the bismuth, which is the main peak of the powder pattern, is not clearly observed, hence the bismuth impurity phase has a preferred orientation to c-axis to minimize the surface energy during the deposition process.

**Figure 4:**
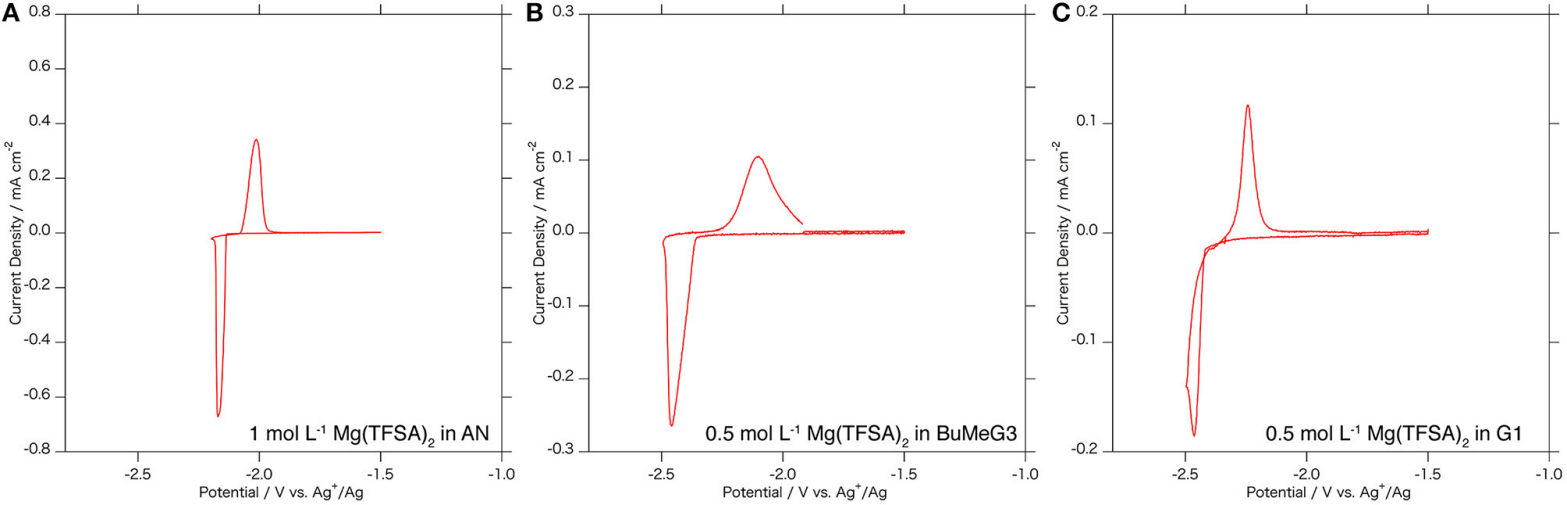
Cyclic voltammograms of the Mg_3_Bi_2_ thin film electrodes in **(A)** 1 mol L^−1^ Mg(TFSA)_2_ in AN solution, **(B)** 0.5 mol L^−1^ Mg(TFSA)_2_ in BuMeG3 solution, and **(C)** 0.5 mol L^−1^ Mg(TFSA)_2_ in G1 solution. Reference electrode is a silver wire immersed in 0.01 mol L^−1^ AgNO_3_ AN solution for **(A)** and 0.01 mol L^−1^ AgTfO solution in BuMeG3/G1 solutions for **(B,C)**. Counter electrode is Pt foil for **(A)** and Mg foil for **(B,C)**. The sweep rate of the voltammetry is 0.1 mV s^−1^.

Figure 4 shows cyclic voltammograms of the Mg_3_Bi_2_ thin film electrodes in various electrolyte solutions: 1.0 mol L^−1^ Mg(TFSA)_2_ in AN, 0.5 mol L^−1^ Mg(TFSA)_2_ in BuMeG3, and 0.5 mol L^−1^ Mg(TFSA)_2_ in dimethoxyethane: G1 (Kishida). The Mg_3_Bi_2_ thin film shows reversible redox reaction in all the three electrolyte solutions. The redox potentials of the Mg_3_Bi_2_ in 0.5 mol L^−1^ Mg(TFSA)_2_ in BuMeG3 and G1 are −2.3 to −2.4 V vs. Ag^+^/Ag, lower than the redox potential in AN electrolyte solution; we suspect it is due to the difference of the Ag^+^/Ag redox potential in the reference electrode corresponding to the solvation properties of AN and the glymes. The broad peaks of the cyclic voltammogram of the BuMeG3 electrolyte solution indicates that the redox reaction is kinetically slow due to the high viscosity of the solution. The overpotential between the anodic and cathodic reaction is ~100 mV, which is slightly higher than that of AN, it is still much lower than the overpotential observed in the deposition/dissolution process of the magnesium metal in these glyme solutions.

### Surface Layer of Mg_3_Bi_2_ Thin Film Electrode

**Figure 5:**
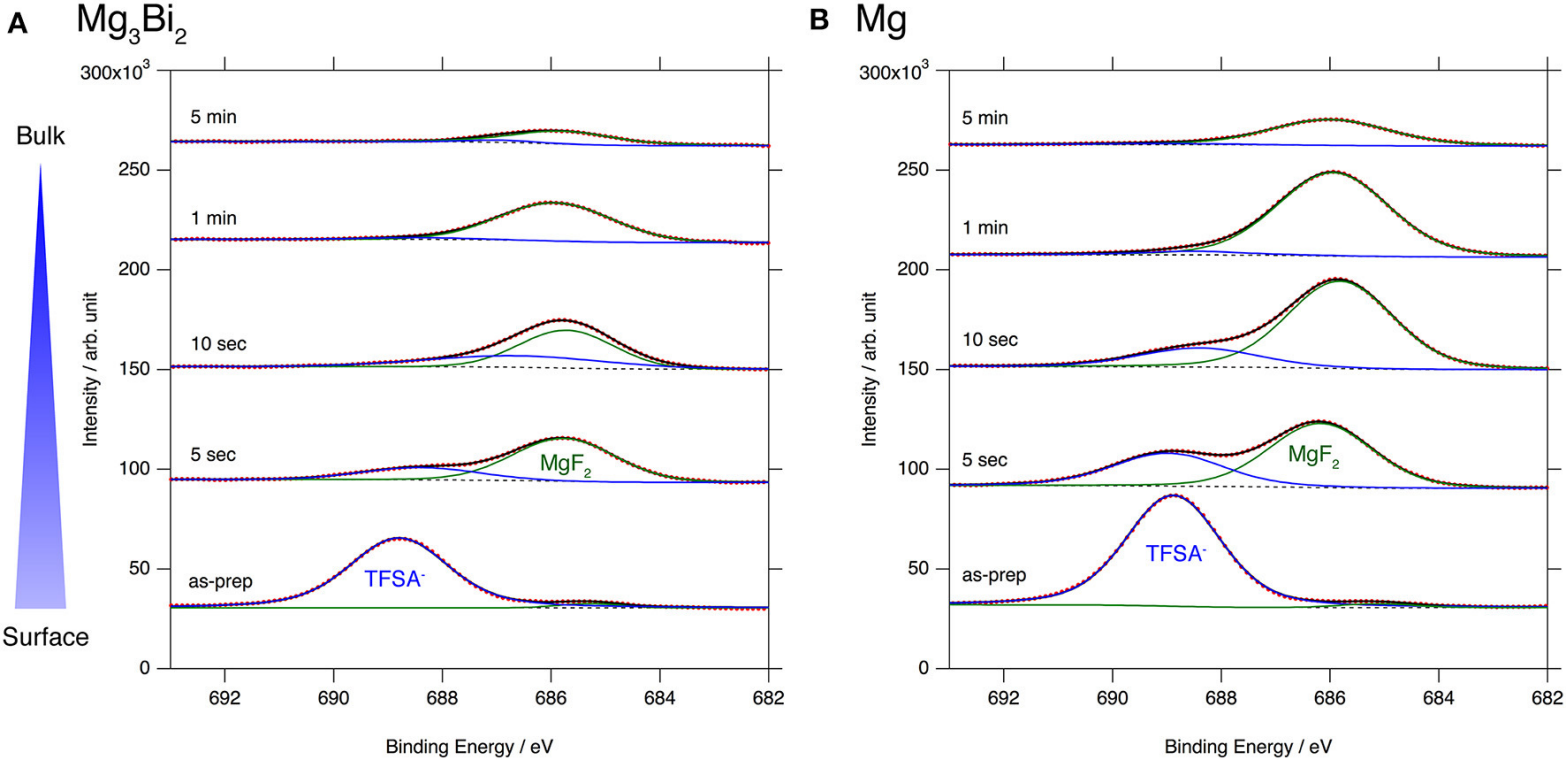
F1s XPS spectra for **(A)** Mg_3_Bi_2_ thin film and **(B)** Mg thin film immersed in 0.5 mol L^−1^ Mg(TFSA)_2_ in BuMeG3 solution for 24 h.

**Figure 6:**
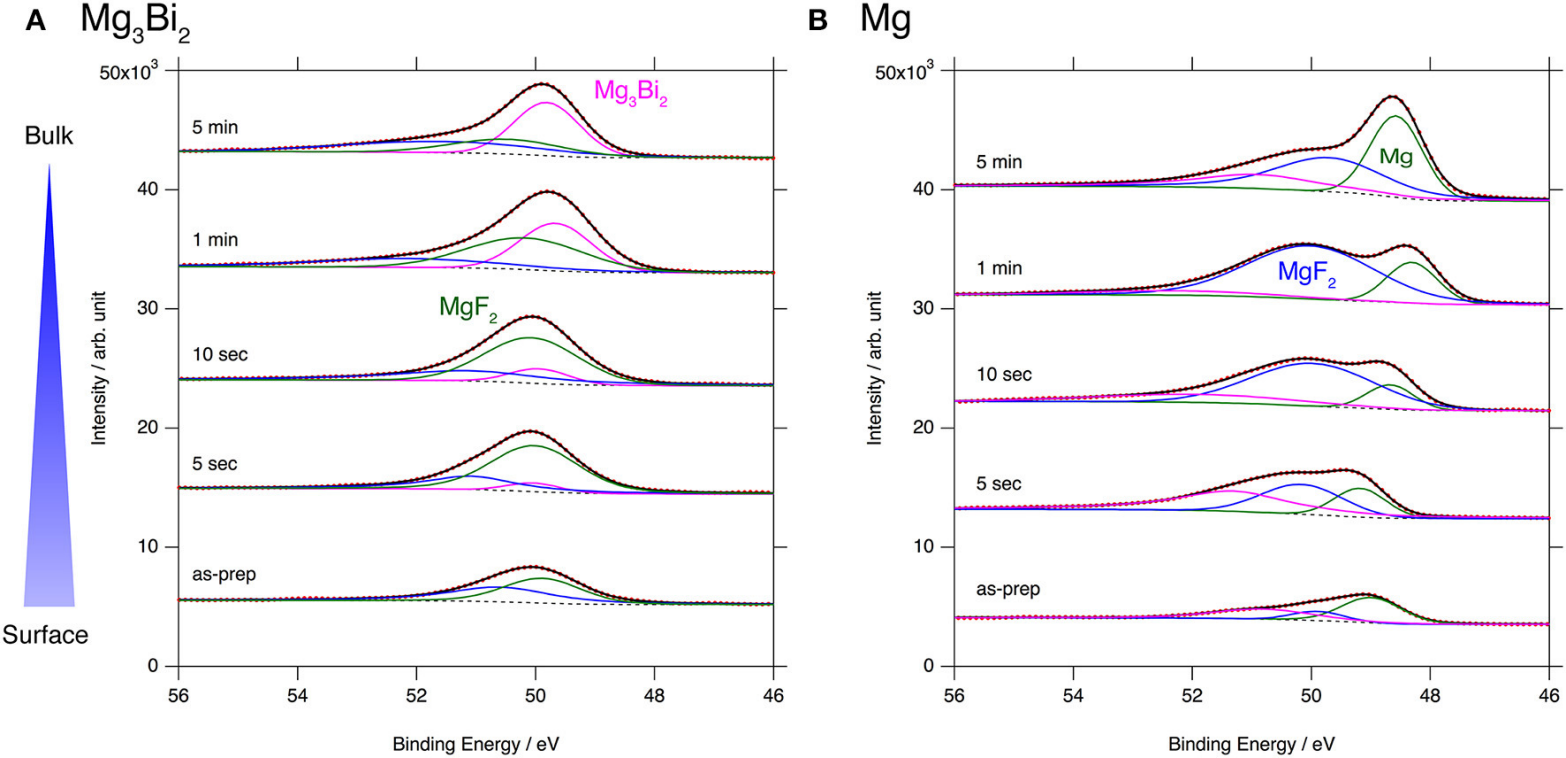
Mg2p XPS spectra for **(A)** Mg_3_Bi_2_ thin film and **(B)** Mg thin film immersed in 0.5 mol L^−1^ Mg(TFSA)_2_ in BuMeG3 solution for 24 h.

The excellent reversibility of the Mg_3_Bi_2_ thin films in the three electrolyte solutions indicates that the surface of the Mg_3_Bi_2_ electrodes are not passivated in these electrolyte solutions. Thus, we performed XPS analyses of the Mg_3_Bi_2_ and the magnesium metal thin film immersed in the electrolyte solution to confirm the absence of the passivation layer. The BuMeG3 solution was chosen for the surface analysis because we previously observed the formation of MgF_2_-based passivation layer at the surface of a commercial magnesium foil (Kuwata et al., 2017). Figure 5 shows F1s XPS spectra of the sputtered Mg_3_Bi_2_ and magnesium metal thin films. Despite our expectation, both of the thin films showed similar spectra to that we observed at the surface of the magnesium foil. The spectra have peaks at 688.8 eV, corresponding to the residue of the TFSA anion at the surface of the electrodes. The TFSA residue gradually decreased and mostly disappeared after 10 seconds of the argon sputtering. Another peak corresponding to the MgF_2_ simultaneously appeared at 685.7 eV during the further sputtering process. The formation of the MgF_2_ layer is a direct evidence of the cathodic decomposition of the TFSA anion in the electrolyte solution. Even though the relatively weak peak intensity of the MgF_2_ on the Mg_3_Bi_2_ suggests the formation of thinner or less dense surface layer, the formation of MgF_2_ proves that the equilibrium potential of the Mg_3_Bi_2_ (0.28 V vs. Mg^2+^/Mg) is still low enough to reduce the TFSA anion. Figure 6 shows Mg2p spectra of the Mg_3_Bi_2_ and the magnesium metal thin films. Even with the fitting result, the removal of the MgF_2_ is not clearly confirmed in the Mg2p XPS spectra of the Mg_3_Bi_2_ thin film shown in Figure 6A, because the peak corresponding to the Mg_3_Bi_2_ and the MgF_2_ are overlapped with each other around 49.8 eV. It shows the Mg atoms in the Mg_3_Bi_2_ phase is positively charged and behave as cations in the solid phase. The Mg2p spectra for the magnesium metal thin film shown in Figure 6B are good reference to understand the structure of the surface layer. A peak corresponding to the magnesium metal appears after 10 seconds of the argon sputtering process at 48.7 eV. Then the peak intensity of the magnesium metal gradually increased during the following sputtering process. Since the decrease of the peak intensity of the MgF_2_ layer is strongly associated with the F1s spectra, we conclude that the main component of the surface layer on these electrodes is MgF_2_. As already discussed in section Electrochemical properties of the Mg_3_Bi_2_ thin film electrodes, the Mg_3_Bi_2_ thin film electrode shows 100 mV of overpotential in the BuMeG3 solution, even with the formation of the MgF_2_ layer. Moreover, the similarity of the surface layer on the Mg_3_Bi_2_ and magnesium metal thin films, suggests that the MgF_2_ does not completely passivate the surface of the both electrodes. Therefore, we think that the origin of the high overpotential of the magnesium deposition/dissolution process in the glyme-based electrolyte solution is not the resistance of the surface layer, but the activation energy of the actual electrode reaction process, which consists of several reaction steps such as adsorption of Mg^2+^ ions, desolvation, electron-transfer, surface, and bulk diffusion of Mg atoms and so forth. Among these reaction steps, we speculate the electron-transfer process significantly contributes to the overpotential, because the biggest difference of the electrode reactions between the Mg_3_Bi_2_ and the magnesium metal, is the oxidation state of the Mg^2+^ ions. As presented in the Mg2p XPS spectra in Figure 6A, the Mg^2+^ ions in the electrolyte solution are not reduced to the Mg atoms in the case of Mg_3_Bi_2_, on the other hand, the Mg^2+^ ions are reduced to the magnesium metal during the electrodeposition process. Further analytical studies are necessary to be conducted to determine the origin of the overpotential.

### Stability Test of the Mg_3_Bi_2_ and Mg_3_Sb_2_

**Figure 7:**
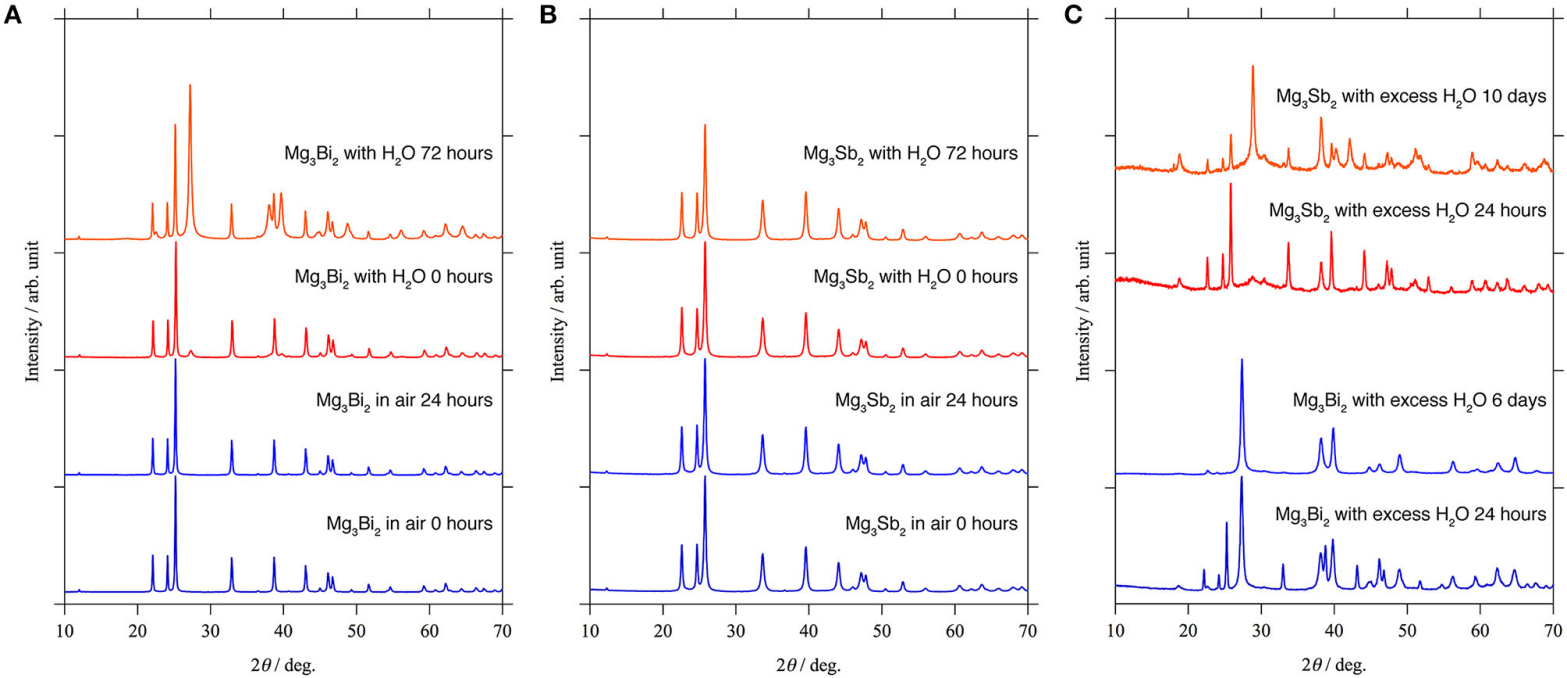
XRD patterns for the stability test: **(A)** Mg_3_Bi_2_ and **(B)** Mg_3_Sb_2_ powder in ambient atmosphere and small amount of water. **(C)** Mg_3_Bi_2_ powder and Mg_3_Sb_2_ powder immersed in the excess amount of water.

Since the XPS results only provide the composition and the oxidation state of the elements, here we investigated the stability of the passivation layer on the Mg_3_Bi_2_ and the Mg_3_Sb_2_ by storing them in ambient atmosphere and water. The XRD patterns of the Mg_3_Bi_2_ and Mg_3_Sb_2_ do not show any changes during the initial 24 h storage in air as shown in Figures 7A,B. It indicates the surfaces of the particles were well passivated. Subsequently we added 0.05 ml of water per sample holder, containing ~0.5 g of the powder sample. Immediately a new peak corresponding to the 012 reflection of bismuth appeared at 27.4° of 2*θ* as shown in Figure 7A. Though no diffraction peaks corresponding to Mg(OH)_2_ are observed, the formation of the bismuth suggests the hydrolysis of the Mg_3_Bi_2_ in the following Equation (1).

(1) $$\begin{array}{l} \text{M}{\text{g}}_{3}\text{B}{\text{i}}_{2}+6{\text{H}}_{2}\text{O}\to 3\text{Mg}{\left(\text{OH}\right)}_{2}+2\text{Bi}+3{\text{H}}_{2}↑ \end{array}$$

We think the missing of the Mg(OH)_2_ is due to the poor crystallinity, because the hydrolysis occurred at room temperature. Furthermore, the hydrolysis also indicates no passivation layer formation at the surface of the Mg_3_Bi_2_. Even though the experimental protocol is not qualified to have quantitative discussions for the reaction kinetics, we think the hydrolysis is a good evidence to prove the passivation-free surface of the Mg_3_Bi_2_. On the other hand, the Mg_3_Sb_2_ never shows any hydrolysis even 72 h after the addition of the water, as if the surface of the Mg_3_Sb_2_ is well-passivated. However, there still remains a question whether the stability is really owing to the passivation layer or the stability of the bulk Mg_3_Sb_2_ phase, considering the chemical similarity between bismuth and antimony. Hence we attempted further acceleration of the hydrolysis by immersing the Mg_3_Bi_2_ and the Mg_3_Sb_2_ powders in excess amount of water. The formation of the Sb is slightly observed after 24 h storage in excess water, and we finally observed the clear evidence of the hydrolysis of the Mg_3_Sb_2_ after 10 days, as shown in Figure 7C. The Mg_3_Bi_2_ obviously showed much faster hydrolysis compared with the Mg_3_Sb_2_ and the hydrolysis is almost completed after 6 days storage. The hydrolysis of the Mg_3_Bi_2_ and the Mg_3_Sb_2_ proves that the formation of the thermodynamically stable intermetallic phase suppress the formation of the passivation layer. As a consequence, an electrochemically active electrode/electrolyte interphase is maintained. We also attribute that the electrochemical activity of the intermetallic anode is highly dependent upon the reaction kinetics, because the hydrolysis of the intermetallics is fundamentally same as the electrochemical reaction. In the hydrolysis process of the intermetallic anodes, the reduction of the proton in the water initiates the oxidation of the Mg_3_Bi_2_ or the Mg_3_Sb_2_ associated with the extraction of Mg^2+^ in the host structure. The Mg_3_Sb_2_ still remains even after the 10 days of the reaction, while the hydrolysis of the Mg_3_Bi_2_ is mostly completed after < 6 days as shown in Figure 7C. It clearly shows the reaction kinetics of the hydrolysis process of the Mg_3_Bi_2_ is much faster than that of the Mg_3_Sb_2_. Even without the quantitative discussion, the fast reaction kinetics of the hydrolysis process well matches with the electrochemical activity of the intermetallic anodes.

### Mg^2+^ Diffusion Pathways in the Mg_3_Bi_2_

The Mg^2+^ diffusion in the bulk active materials is one of the key parameters which determines the reaction kinetics of the magnetization/demagnetization process, here we investigated the diffusion pathways of Mg^2+^ in the Mg_3_Bi_2_ and the Mg_3_Sb_2_. The bond valence sum (BVS) mapping is one methodology to visualize the pathways of the mobile ions in the crystal structure. The bond-valence parameters for Mg_3_Bi_2_ and Mg_3_Sb_2_ were calculated by following equation (Brese and O'Keeffe, 1991).

(2) $$\begin{array}{l} Ro=bln\left[V_{ij}/\Sigma_{j}\text{exp}\left({-d}_{ij}/b\right)\right] \end{array}$$

**Figure 8:**
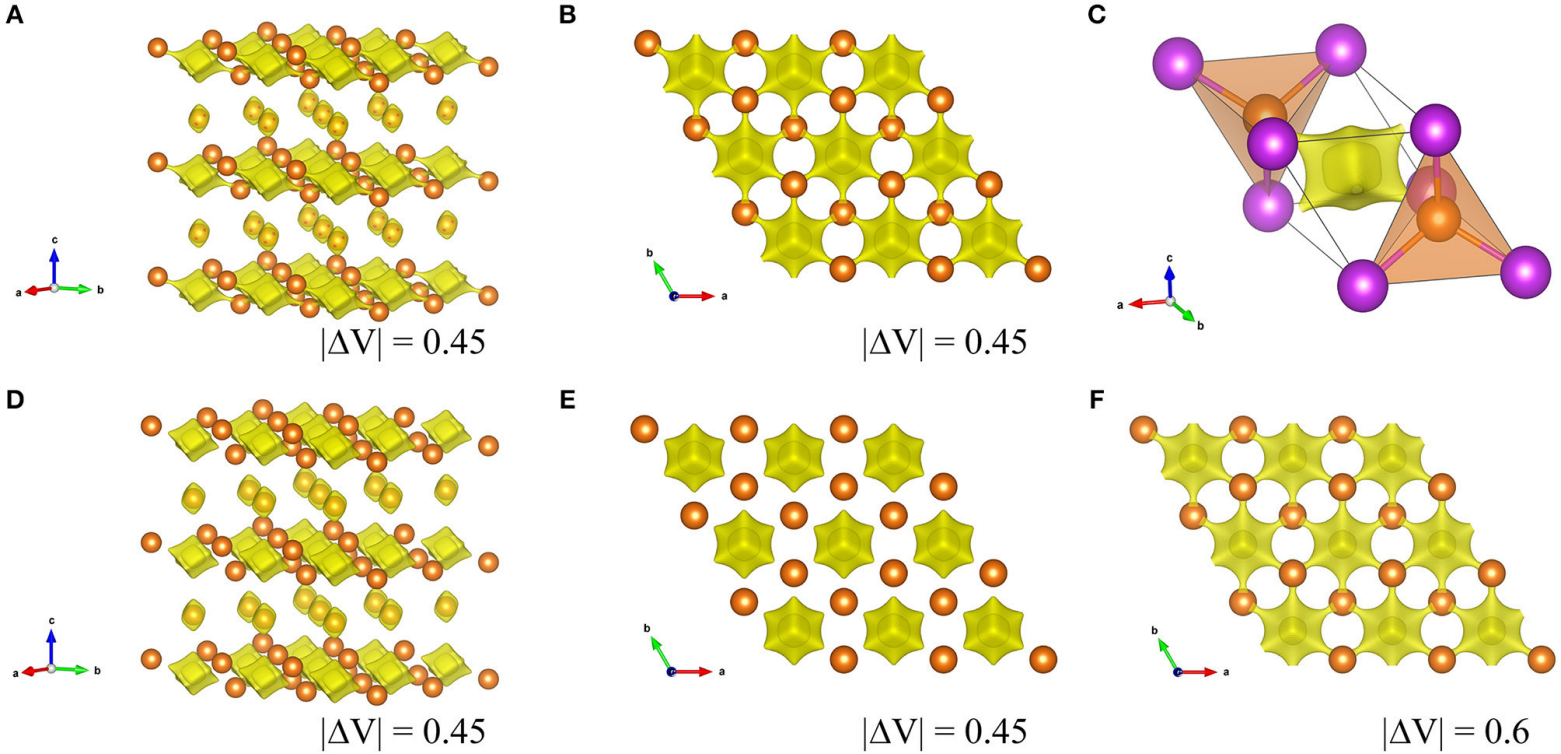
Visualized Mg^2+^ diffusion pathways by BVS mapping in Mg_3_Bi_2_ and Mg_3_Sb_2_. **(A)** overview of Mg_3_Bi_2_, **(B)** the 2D diffusion pathways in 2*d* tetrahedral sites in Mg_3_Bi_2_ from c-axis, and **(C)** an enlarged image of two MgBi_4_ tetrahedra with an interstitial octahedron, **(D)** overview of Mg_3_Sb_2_, **(E)** 2*d* tetrahedral sites in Mg_3_Sb_2_ from c-axis **(F)** 2*d* tetrahedral sites in Mg_3_Sb_2_ from c-axis at cut off |ΔV| = 0.6.

Here *b* is commonly taken to a constant value 0.37 Å. *V*_*ij*_ is sum of valences of given atoms *i* and *d*_*ij*_ is bond length from refinement results. The calculated *Ro* value for the Mg_3_Bi_2_ and the Mg_3_Sb_2_ are 2.687 and 2.617, respectively. Figure 8 shows the BVS mapping of the Mg_3_Bi_2_ and the Mg_3_Sb_2_ using the refined structure model with discussed in Electrochemical properties of the Mg_3_Bi_2_ and Mg_3_Sb_2_ composite electrode. The BVS mappings of the Mg_3_Bi_2_ clearly show 2D layered diffusion pathways connecting the 2*d* tetrahedral sites at cut off value: |ΔV| = 0.45 as shown in Figures 8A,B. Figure 8C shows a migration path between the two tetrahedral sites of the Mg_3_Bi_2_. The Mg^2+^ ion at the tetrahedral site is preferred to migrate to the next tetrahedral site, via the interstitial octahedral site. The estimated migration path is in good agreement with the proposed diffusion path model for Mg^2+^ by DFT-NEB simulations (Rong et al., 2015). Jung et al. also reported that the migration barrier in between two tetrahedral sites of Mg_3_Bi_2_ is 0.30 eV, suggesting fast Mg^2+^ mobility in the Mg_3_Bi_2_ phase (Jung and Han, 2018). In addition, once the vacancy is formed, the fast migration of the Mg^2+^ from the octahedral site to the vacancy at the tetrahedral site is also expected (Lee et al., 2018). Even though the BVS mapping is not as accurate as DFT modeling, the estimated Mg^2+^ diffusion pathways are qualitatively informative to discuss the fast Mg^2+^ mobility in the solid phase. We also performed the BVS mapping of the Mg_3_Sb_2_ as shown in Figures 8D,E. At glance, the estimated diffusion pathways look similar to the mapping of the Mg_3_Bi_2_, however all the preferred diffusion path at interstitial octahedral sites are isolated at cut off value: |ΔV| = 0.45, as seen in Figure 8E. Figure 8F shows the 2D layered diffusion pathways in the Mg_3_Sb_2_ formed at cut off value: |ΔV| = 0.6. The higher cut off value indicates that the higher migration barrier of the Mg^2+^ ions in the Mg_3_Sb_2_ (Fedotov et al., 2018). The BVS mapping results suggests that the Mg^2+^ migration in Mg_3_Bi_2_ is qualitatively faster than in Mg_3_Sb_2_. Though we still need further validation analyses, all the results are consistent with the contribution of the fast Mg^2+^ mobility to the excellent electrochemical properties of the Mg_3_Bi_2_.

## Conclusion

In the present study, we investigated the electrochemical properties of the magnesium-based intermetallic compounds as alternative anode active material for magnesium batteries. Even though the Mg_3_Bi_2_ and the Mg_3_Sb_2_ have same crystal structure, the electrochemical activity of the Mg_3_Sb_2_ was almost negligible, while the Mg_3_Bi_2_ showed highly reversible redox reaction at −2.1 V vs. Ag^+^/Ag. The XPS analyses of the Mg_3_Bi_2_ thin films showed formations of the MgF_2_-based surface layer, which is the reduction product of the TFSA anion and also observed at the surface of the magnesium metal thin film. On the other hand, the excellent electrochemical activity of Mg_3_Bi_2_ in three electrolyte solutions: 1 mol L^−1^ Mg(TFSA)_2_ in AN, 0.5 mol L^−1^ Mg(TFSA)_2_ in BuMeG3, and 0.5 mol L^−1^ Mg(TFSA)_2_ in DME, suggests that the surface of the Mg_3_Bi_2_ is not passivated even with the formation of the MgF_2_ layer. We also investigated the stability of the Mg_3_Bi_2_ and the Mg_3_Sb_2_ in ambient atmosphere and in water. The stability tests of the intermetallics revealed that the formation of the intermetallics is an effective way to avoid the passivation of the anode surface. The results also suggested the reversibility of the intermetallic anode is not only dependent on the passivation-free surface, but on the fast reaction kinetics. The BVS mapping showed that the Mg^2+^ conduction pathway among tetrahedral sites well matches with the predicted fast Mg^2+^ diffusion path by DFT modeling. Here we conclude that the formation of the intermetallics is effective strategy to avoid the passivation of anode surface, which hinders the reversible redox reaction. The fast Mg^2+^ ion mobility in the solid phase is another key parameter to obtain the good reversibility applicable to a practical battery system.

## Author Contributions

MaM and HK designed the research approach and experiments. HK carried out the synthesis work. MaM, HK, and DM carried out the structural analyses and the surface analyses. HK, NI, and MiM carried out the electrochemical analyses. MaM wrote the manuscript and all authors discussed the final manuscript.

### Conflict of Interest Statement

The authors declare that the research was conducted in the absence of any commercial or financial relationships that could be construed as a potential conflict of interest.
